# A Novel tRNA-Derived Fragment, tRF^GlnCTG^, Regulates Angiogenesis by Targeting Antxr1 mRNA

**DOI:** 10.3390/ijms241914552

**Published:** 2023-09-26

**Authors:** Qiuyang Chen, Linyuan Shen, Tianci Liao, Yanhao Qiu, Yuhang Lei, Xingyu Wang, Lei Chen, Ye Zhao, Lili Niu, Yan Wang, Shunhua Zhang, Li Zhu, Mailin Gan

**Affiliations:** 1 Farm Animal Genetic Resource Exploration and Innovation Key Laboratory of Sichuan Province, Sichuan Agricultural University, Chengdu 611130, China; 2021202021@stu.sicau.edu.cn (Q.C.); shenlinyuan@sicau.edu.cn (L.S.); 2020202019@stu.sicau.edu.cn (T.L.); 2021202043@stu.sicau.edu.cn (Y.Q.); 2022202014@stu.sicau.edu.cn (Y.L.); 2022202034@stu.sicau.edu.cn (X.W.);; 2 Key Laboratory of Livestock and Poultry Multi-omics, Ministry of Agriculture and Rural Affairs, College of Animal and Technology, Sichuan Agricultural University, Chengdu 611130, China

**Keywords:** tRNA-derived fragment, angiogenesis, proliferation, Antxr1, C166 cells

## Abstract

As a novel non-coding RNA with important functions corresponding to various cellular stresses, the function of tRFs in angiogenesis remains unclear. Firstly, small RNA sequencing was performed on normal and post-muscle injury mouse tibialis anterior muscle to identify and analyse differentially expressed tRF/tiRNA. tRNA GlnCTG-derived fragments (tRF^GlnCTG^) were found to be overexpressed in high abundance in the damaged muscle. Subsequent in vitro experiments revealed that the overexpression of tRF^GlnCTG^ suppressed the vascular endothelial cells’ viability, cell cycle G1/S transition, proliferation, migration, and tube-formation capacity. Similarly, in vivo experiments showed that the tRF^GlnCTG^ decreased the relative mRNA levels of vascular endothelial cell markers and pro-angiogenic factors and reduced the proportion of CD31-positive cells. Finally, luciferase activity analysis confirmed that the tRF^GlnCTG^ directly targeted the 3′UTR of Antxr1, leading to a significant reduction in the mRNA expression of the target gene. These results suggest that tRF^GlnCTG^ is a key regulator of vascular endothelial cell function. The results provide a new idea for further exploration of the molecular mechanisms that regulate angiogenesis.

## 1. Introduction

The formation of new blood vessels is a critical step in tissue organ morphogenesis and tissue remodelling [1]. Angiogenesis is defined as the growth of new capillaries on pre-existing blood vessels [2]. The regulation of angiogenesis has been extensively studied for the treatment of ischaemic diseases, wound healing, and tissue regeneration [3]. Studies suggest that muscle regeneration defects may be attributed to damaged blood vessels [4]. It is now widely accepted that angiogenesis in skeletal muscle is dependent on pro-and anti-angiogenic molecules that regulate endothelial cell proliferation and migration [5,6]. Available evidence suggests that functional tissue regeneration requires functional angiogenesis [7,8].

Transfer RNA (tRNA)-derived fragments (tRFs) are small single stranded RNA molecules derived from pre-tRNAs and mature tRNAs of sizes ranging from 18 to 50 nt [9,10,11]. A growing body of research suggests that tRFs play multiple biological roles, including functioning as microRNAs (miRNAs) to regulate translation, gene expression, and cellular stress responses [12]. However, as a novel non-coding RNA with important functions corresponding to various cellular stresses [13], the function of tRFs in angiogenesis remains unclear.

Antxr1, also known as Tem8, is a highly conserved cell surface protein that is a marker of tumour-associated endothelial cells versus normal endothelial cells in colorectal cancer [14]. Antxr1 is important for promoting tumour angiogenesis, and it has been reported that Antxr1 blockade inhibits pathologic angiogenesis [15] and that overexpression of Antxr1 stimulates the migration of endothelial cells [16]. DNA vaccines against Antxr1 can inhibit tumour angiogenesis [17]. However, there are still few studies reporting the effect of Antxr1 on angiogenesis in muscle tissue, much less its regulation by tRFs.

This study aimed to investigate the relationship between tRNA-derived fragments and angiogenesis in muscle; we found that tRF^GlnCTG^, a transfer RNA (tRNA)-derived fragment (tsRNA) that is highly expressed 3 days after muscle injury, inhibits angiogenesis in vitro by suppressing the proliferation, migration, and tube formation of the C166 vascular endothelial cells of mice. We further verified that tRF^GlnCTG^ inhibits angiogenesis by targeting Antxr1, and that the knockdown of Antxr1 similarly inhibits mice’s C166 vascular endothelial cell proliferation, migration, and tube formation in vitro. By overexpressing tRF^GlnCTG^ in vivo, we found that it inhibited angiogenesis at 7 days, 14 days after muscle injury. Our results reveal a potential role for tRF^GlnCTG^, an emerging small non-coding RNA, in tissue repair through the inhibition of angiogenesis.

## 2. Results

### 2.1. Muscle Vessels Were Damaged and tRF^GlnCTG^ Expression Increased 3 Days after Muscle Injury

**Figure 1:**
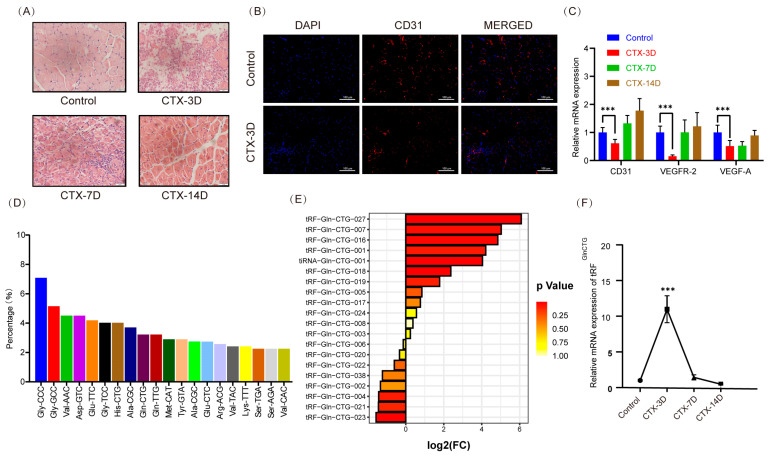
Muscle vessels were damaged and tRF^GlnCTG^ expression increased 3 days after muscle injury. (**A**) Representative HE-staining of mouse tibialis anterior muscle 3 days after PBS injection in control group and 3, 7, and 14 days after CTX injection. Scale: 1 bar represents 20 μm. (**B**) Immunofluorescence results of the vascular endothelial cell marker CD31 in mouse tibialis anterior muscle of control group and 3 days after CTX injury. Scale: 1 bar represents 100 μm. (**C**) mRNA levels of angiogenesis-related markers in mouse tibialis anterior muscle at 3 days, 7 days, and 14 days after CTX injury. (**D**) Top 20 ranking of tsRNAs produced by different tRNAs. (**E**) Histogram of Log2(FC) values versus *p* values for tsRNAs produced by tRNA Gln CTG. (**F**) RT-PCR analysis of tRF^GlnCTG^ levels in mouse tibialis anterior muscle at 3 days, 7 days, and 14 days after CTX injury (*n* = 3 per group). The values are expressed as Mean ± SD of biological triplicates. ***, *p* < 0.001 using unpaired two-tailed Student’s *t* test.

Initially, 20 μM of cardiotoxin (CTX) in 50 μL was injected into the tibialis anterior muscle of 8-week-old male mice, while an equal amount of PBS was injected into the control group. The tibialis anterior muscle was collected at three, seven, and fourteen days after the injection, and the H&E staining revealed that the muscle was most severely damaged 3 days after CTX treatment (Figure 1A). The CD31 immunofluorescence showed a reduced number of positive cells in the CTX-treated group compared to the control group (Figure 1B). Subsequently, we examined the mRNA levels of relevant vascular endothelial cell markers and found a significant reduction 3 days after muscle injury (Figure 1C, *p* < 0.05), indicating that angiogenesis in the muscle was impaired at this time-point.

It has been reported that the tRNA-derived fragment tRF^GlnCTG^ promotes the proliferation of vascular smooth muscle cells [18]. Using small RNA sequencing, we found a total of 621 tsRNAs differentially expressed 3 days after muscle injury, derived from 48 tRNAs. Among them, 3.22% of the total tsRNAs were derived from tRNA Gln CTG, ranking ninth (Figure 1D). Next, we found that tRF-Gln-CTG-027 (length 19 nt) had the largest fold change in the tsRNAs produced by tRNA GlnCTG. Also, we found that the expression of tRF^GlnCTG^ significantly increased 3 days after muscle injury (Figure 1E), and the real-time PCR results confirmed that the expression level of tRF^GlnCTG^ in mouse tibialis anterior muscle significantly increased 3 days after muscle injury, and then gradually decreased on days 7 and 14 (Figure 1F, *p* < 0.05). Therefore, we are interested in further investigating the potential regulatory role of tRF^GlnCTG^ on angiogenesis after muscle injury.

### 2.2. Overexpression of tRF^GlnCTG^ Inhibits Angiogenesis by Inhibiting C166 Mouse Vascular Endothelial Cells Proliferation, Migration, and Tube Formation In Vitro

**Figure 2:**
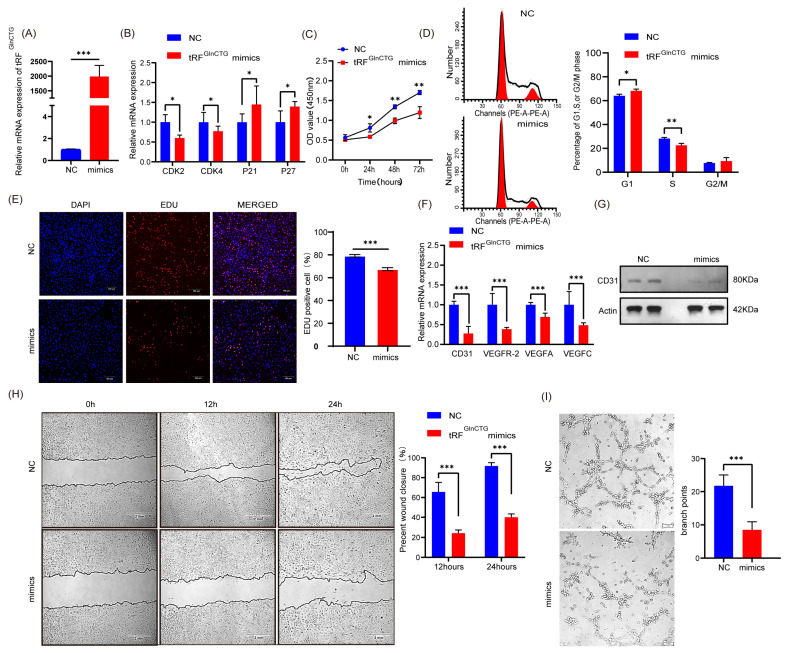
tRF^GlnCTG^ inhibits angiogenesis by inhibiting C166 mouse vascular endothelial cells proliferation, migration, and tube formation in vitro. (**A**) Fold change of tRF^GlnCTG^ after transfection mimics compared to negative controls. (**B**) Relative mRNA levels of cell cycle-related markers (CDK2, CDK4, P21, and P27) in transfected cells. (**C**) Cell viability of C166 mouse vascular endothelial cells at different time-points after overexpression of tRF^GlnCTG^. (**D**) Flow cytometric analysis was performed to detect the cell cycle of C166 mouse vascular endothelial cells after overexpression of tRF^GlnCTG^. (**E**) The 5-Ethynyl-2′-deoxyuridine (Edu) kit is used to detect the proliferative capacity of C166 mouse vascular endothelial cell cells after overexpression of tRF^GlnCTG^. Scale: 1 bar represents 100 μm. (**F**,**G**) mRNA and protein levels of angiogenesis-related markers in the vascular endothelium of C166 mice after overexpression of tRF^GlnCTG^. β-actin was used as a loading control. (**H**) Cell migration capacity of C166 mouse vascular endothelial cells after overexpression of tRF^GlnCTG^ was analysed through wound healing. Scale: 1 bar represents 2 mm. (**I**) C166 mouse vascular endothelial cells after overexpression of tRF^GlnCTG^ were digested and redistributed into 96-well plates coated with Matrigel for tube-formation assays. Representative images of tube formation by C166 mouse vascular endothelial cells are shown. Scale: 1 bar represents 1 mm. The values are expressed as Mean ± SD of biological triplicates. *, *p* < 0.05; **, *p* < 0.01; and ***, *p* < 0.001 using unpaired two-tailed Student’s *t* test.

Next, we investigated the role of tRF^GlnCTG^ on angiogenesis by transfecting tRF^GlnCTG^ mimics into the C166 vascular endothelial cells of mice and examining their expression levels. We observed a significant increase in tRF^GlnCTG^ expression in the transfected cells (Figure 2A, *p* < 0.05). Using real-time PCR, we found that the relative mRNA expression levels of CDK2 and CDK4, which promote the G1/S transition in the cell cycle, significantly decreased, while the relative mRNA levels of P21 and P27, inhibitors of CDK molecules, significantly increased (Figure 2B, *p* < 0.05). The CCK-8 assay suggested that the viability of vascular endothelial cells had lowered after tRF^GlnCTG^-overexpression (Figure 2C, *p* < 0.05). Additionally, the flow cytometric assay showed that the G1-S phase transition of vascular endothelial cells was inhibited by the transfection with mimics, meaning that more cells remained in the G1 phase and failed to enter the S phase (Figure 2D, *p* < 0.05). Furthermore, the EDU assay showed that the number of positive cells in the proliferative phase significantly decreased after mimics treatment, with an 11.7% decrease in the proportion of EDU-positive cells in the mimics-treated group (Figure 2E, *p* < 0.05). All these results demonstrated that tRF^GlnCTG^ inhibited the proliferative ability of vascular endothelial cells in mice.

To further verify the effect of tRF^GlnCTG^ on angiogenesis, we examined the relative mRNA level of vascular endothelial cells markers and pro-angiogenic factors after transfection with mimics. We found that the mRNA levels of these markers and factors significantly decreased, and that the protein levels of vascular endothelial cell markers had also significantly reduced (Figure 2F,G, *p* < 0.05). Moreover, the wound-healing assay revealed a significant decrease in the vascular endothelial cells’ migration capacity after mimics treatment; the rate of wound healing decreased by 41.4% at 12 h and 51.8% at 24 h in the mimics-treated group (Figure 2H, *p* < 0.05). Most importantly, the tube-formation assay verified that the endothelial cells’ tubular structure formation was significantly inhibited by the mimics treatment (Figure 2I, *p* < 0.05). Collectively, these results demonstrate that tRF^GlnCTG^ negatively regulates angiogenic capacity by inhibiting multiple biological events.

### 2.3. tRF^GlnCTG^ Directly Targets the 3′UTR of Antxr1 and Knockdown of Antxr1 Inhibits Angiogenesis by Inhibiting C166 Mouse Vascular Endothelial Cell Proliferation, Migration, and Tube Formation In Vitro

**Figure 3:**
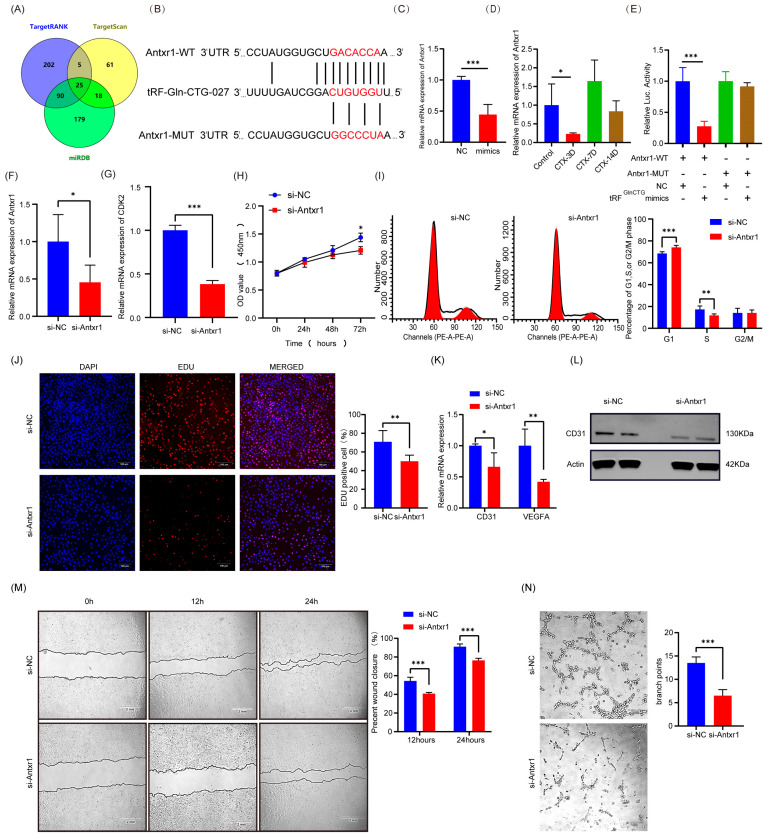
tRF^GlnCTG^ directly targets the 3′UTR of Antxr1 and knockdown of Antxr1 inhibits angiogenesis by inhibiting C166 mouse vascular endothelial cell proliferation, migration, and tube formation in vitro. (**A**) Venn diagrams of tRF^GlnCTG^ target genes predicted by three prediction sites (TargetScan, TargetRank, and miRDB). (**B**) Theoretically predicted binding site between tRF^GlnCTG^ and Antxr1. (**C**,**D**) mRNA levels of Antxr1 in C166 cells 24 h after transfection with NC and tRF^GlnCTG^ mimics, and in WT TA muscle 3 days after CTX injury. (**E**) The Antxr1-3′UTR-WT or mutant reporter plasmid was co-transfected into 293T cells with tRF^GlnCTG^ mimics or NC. Luciferase activity of Antxr1-3′UTR-WT was significantly decreased by tRF^GlnCTG^ in cells. (**F**) Knockdown of Antxr1 transfection efficiency in C166 mouse vascular endothelial cells using small interfering RNA (siRNA). (**G**) Relative mRNA levels of CDK2 in C166 mouse vascular endothelial cells after knockdown of Antxr1. (**H**) Cell viability of C166 mouse vascular endothelial cells at different time-points after knockdown of Antxr1. (**I**) Flow cytometric analysis was performed to detect the cell cycle of C166 mouse vascular endothelial cells after knockdown of Antxr1. (**J**) The 5-ethynyl-2′-deoxyuridine (Edu) kit was performed to detect the proliferative capacity of C166 mouse vascular endothelial cell cells after knockdown of Antxr1. Scale: 1 bar represents 100 μm. (**K**,**L**) mRNA and protein levels of angiogenesis-related markers in C166 mouse vascular endothelial cells after knockdown of Antxr1. β-actin was used as a loading control. (**M**) Cell migration capacity of C166 mouse vascular endothelial cells after knockdown of Antxr1 was analysed through wound healing. Scale: 1 bar represents 2 mm. (**N**) Knockdown of Antxr1 after C166 mouse vascular endothelial cells were digested and redistributed into 96-well plates coated with Matrigel for tube-formation assays. Representative images of tube formation by C166 mouse vascular endothelial cells are shown. Scale: 1 bar represents 1 mm. The values are expressed as Mean ± SD of biological triplicates. *, *p* < 0.05; **, *p* < 0.01; and ***, *p* < 0.001 using unpaired two-tailed Student’s *t* test.

In order to investigate the molecular mechanisms underlying the anti-angiogenic effect of tRF^GlnCTG^, we used the seed sequence of tRF^GlnCTG^ for target gene prediction in three algorithms: TargetScan, TargetRank, and miRDB (Figure 3A). Further analysis using RNAhybrid revealed a high degree of complementarity between tRF^GlnCTG^ and the 3′UTR of Antxr1, a tumour endothelial marker strongly associated with angiogenesis (Figure 3B). Subsequently, we observed a significant reduction in the Antxr1 mRNA levels in both the CTX-treated tissues and the vascular endothelial cells that had been transfected with tRF^GlnCTG^ mimics, as determined by the real-time PCR (Figure 3C,D, *p* < 0.05). Dual-luciferase reporter assays confirmed that the overexpression of tRFGlnCTG significantly decreased the fluorescence intensity of the Antxr1 wild-type luciferase reporter plasmid, but not the mutant plasmid (Figure 3E, *p* < 0.05). These results demonstrate that tRF^GlnCTG^ directly binds to the 3′UTR of Antxr1 in order to negatively regulate its function.

We then designed a small interfering RNA (siRNA)-mediated Antxr1-knockdown assay to further validate the regulatory role of tRF^GlnCTG^ on Antxr1 and to investigate the role of the tRF^GlnCTG^–Antxr1 axis in the regulation of angiogenesis. After the successful knockdown of the mRNA levels of Antxr1 in the vascular endothelial cells (Figure 3F, *p* < 0.05), we found that the relative mRNA expression level of CDK2 significantly decreased (Figure 3G, *p* < 0.05). The CCK-8 assay suggested that the viability of vascular endothelial cells had lowered after the knockdown of Antxr1 (Figure 3H, *p* < 0.05). The flow cytometry results showed that more cells had remained in G1 and had failed to enter the S phase after the knockdown of Antxr1 (Figure 3I, *p* < 0.05). Additionally, the EDU assay showed that the number of positive cells in the proliferative phase significantly decreased after the knockdown of Antxr1, with a 20.84% decrease in the proportion of EDU-positive cells in the Antxr1 knockdown group (Figure 3J, *p* < 0.05). All these results suggest that the knockdown of Antxr1 inhibits the proliferative capacity of mouse vascular endothelial cells, in a manner that is consistent with the effects of the overexpression of tRF^GlnCTG^.

We also confirmed the effect of the knockdown of Antxr1 on angiogenesis. We quantified the relative mRNA levels of vascular endothelial cell marker genes and pro-angiogenic factors after the knockdown of Antxr1 and found that they had significantly reduced, alongside the protein levels of the vascular endothelial cell marker CD31 (Figure 3K,L, *p* < 0.05). The wound-healing assay revealed a significant decrease in the vascular endothelial cells’ migration capacity after the knockdown of Antxr1: the rate of wound healing decreased by 13.2% at 12 h and 17.6% at 24 h in the Antxr1-knockdown group (Figure 3M, *p* < 0.05). The tube-formation assay verified that the endothelial cells’ tubular structure formation was significantly inhibited by the knockdown of Antxr1 (Figure 3N, *p* < 0.05). These results indicate that the knockdown of Antxr1 has a negative regulatory effect on angiogenesis, which is consistent with the overexpression of tRF^GlnCTG^.

### 2.4. Overexpression of tRF^GlnCTG^ Inhibits Angiogenesis In Vivo

**Figure 4:**
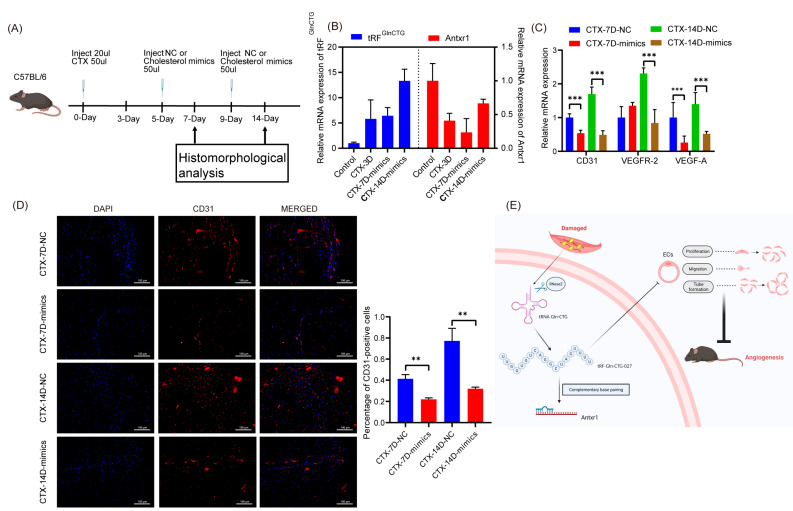
Overexpression of tRF^GlnCTG^ inhibits angiogenesis in vivo. (**A**) A technical route to overexpress tRF^GlnCTG^ in vivo. Mouse tibialis anterior muscle was injected with NC or cholesterol-modified mimics 5 days and, again, 9 days after CTX injury to maintain high levels of tRF^GlnCTG^ expression. (**B**) Fold change of tRF^GlnCTG^ and its target gene Antxr1 7 days and 14 days after CTX injury by injection of NC or cholesterol-modified mimics into the tibialis anterior muscles of mice. (**C**) Expression levels of angiogenesis-related markers 7 days and 14 days after CTX injury in mice injected with NC or cholesterol-modified mimics in the tibialis anterior muscle. (**D**) Immunofluorescence results of CD31 7 days and 14 days after CTX injury in mice injected with NC or cholesterol-modified mimics in the tibialis anterior muscle. Scale: 1 bar represents 100 μm. (**E**) Model for high expression of tRF^GlnCTG^ that regulates angiogenesis by targeting Antxr1 after muscle injury. The values are expressed as Mean ± SD of biological triplicates. **, *p* < 0.01; and ***, *p* < 0.001 using unpaired two-tailed Student’s *t* test.

To determine the effect of tRF^GlnCTG^ on angiogenesis during muscle repair in vivo, we proceeded with the following steps. Firstly, we obtained muscle-injured mice and injected either NC or cholesterol-modified mimics 5 days and 9 days after CTX injection to maintain a high expression of tRF^GlnCTG^ in the mice’s tibialis anterior muscles. Secondly, we collected the tibialis anterior muscles from the mice 7 days and 14 days after CTX injection (Figure 4A). Using real-time PCR, we found that the cholesterol-modified mimics group maintained a high expression of tRF^GlnCTG^ for 7–14 days after muscle injury, while the relative mRNA levels of the target gene Antxr1 remained low; these results demonstrated that we had successfully overexpressed the tRF^GlnCTG^ in the mice’s muscles (Figure 4B, *p* < 0.05).

Next, to investigate the effect of the overexpression of tRF^GlnCTG^ on angiogenesis in tissues, we quantified the markers of vascular endothelial cells, as well as the pro-angiogenic factors, and showed that their expression levels were lower in the cholesterol-modified mimic group than in the control group 7 days and 14 days after muscle injury (Figure 4C, *p* < 0.05). Most importantly, the tissue CD31’s immunofluorescence results showed that the cholesterol-modified mimics group had a lower proportion of CD31-positive cells 7 days and 14 days after muscle injury compared to the control group (Figure 4D, *p* < 0.05). All these results demonstrate that the overexpression of tRF^GlnCTG^ inhibits angiogenesis in mouse muscle in vivo.

## 3. Discussion

In this study, we focused on the functional effects of the tRNA-derived fragment tRF^GlnCTG^ on C166 mouse vascular endothelial cells after muscle injury. Through high-throughput sequencing, we found that the high expression of tRF^GlnCTG^ 3 days after muscle injury is closely associated with angiogenesis. The overexpression of tRF^GlnCTG^ in vitro inhibited the proliferation, migration, and tube formation of vascular endothelial cells, and the knockdown of its target gene, Antxr1, had the same result. The overexpression of tRF^GlnCTG^ in vivo similarly inhibits angiogenesis following muscle injury. In summary, the tRF^GlnCTG^–Antxr1 signalling pathway may provide new ideas and therapeutic strategies for the study of muscle angiogenesis. These findings imply that tRFs may act as novel epigenetic molecules in the regulation of angiogenesis.

Acute muscle injury induced by CTX has been shown to be characterised by the death of the microvascular system, which is consistent with the impaired angiogenesis observed in the damaged muscle tissue in our study [19]. The regulation of angiogenesis by non-coding RNAs has been well documented: for example, microRNA-92a-3p inhibits endothelial cell tube formation, cell migration, and wound healing by targeting SGK3 [20]. tRNA derivatives have complex and diverse regulatory mechanisms, and some tRNA derivatives and miRNAs have been shown to have similar functions in inhibiting mRNA translation [21,22]. In this study, tRF^GlnCTG^ targeted Antxr1 in a miRNA-like manner to inhibit angiogenesis. In line with this study, previous studies have found that 5′tiRNA-Gly-CCC promotes muscle regeneration by targeting Tgfbr1 through an inflammatory response [23]. During ocular angiogenesis, tRF-1001 regulates endothelial angiogenic effects via tRF-1001/METTL3/RBPJ-MAML1 signalling [24].

There are still some limitations to our study that need to be addressed in future research. Firstly, we only focused on the tsRNAs produced by tRNA GlnCTG, while there are still large numbers of differentially expressed tsRNAs whose functions on muscle regeneration, as well as angiogenesis, deserve further attention and study. Secondly, we found that the overexpression of tRF^GlnCTG^ and the knockdown of its target gene Antxr1 differed in their effects on the expression of some genes, e.g., CDK4 and VEGFR-2. This is because we used a seed sequence alignment method to predict the target genes. It is unknown whether the tRF^GlnCTG^ inhibits angiogenesis through other mechanisms, such as protein interactions and chromatin regulation, and its binding relationships with multiple other target genes also need to be further investigated. Our study provides preliminary evidence for the relationship between tRFs and angiogenesis, but more experiments are needed to verify this relationship. These limitations will be addressed in our future research.

## 4. Materials and Methods

### 4.1. Animals

The animal study protocol was approved by the Sichuan Agricultural University Ethics and Welfare Committee (No.20210156) on 2 July 2022. Eight-week male C57BL/6J mice were purchased from Chengdu Dossy Experimental animals Co, Ltd. (Chengdu, China). The mice had free access to food and water throughout the experimental period (14 days). Snake venom cardiotoxin (CTX) was purchased from Solarbio (Beijing, China). The right tibialis anterior (TA) muscle of the experimental group (*n* = 21) was injected with CTX (50 μL and 20 μM phosphate-buffered saline), and three mice were killed three, seven, and fourteen days after CTX injection, respectively. After five and nine days from the CTX injection, twelve mice were injected with NC or with a cholesterol-modified analogue (50 μL and 20 μM, respectively) (Shanghai Gene Pharmaceutical Co., Ltd., Shanghai, China) into the right TA muscle and six mice (three each of NC and cholesterol-modified analogue) were sampled on day 7 and day 14, respectively. The control group was injected with an equal amount of PBS (*n* = 6) and the samples were collected 3 days after the simultaneous injection with the CTX. The mice’s TA muscles were excised, and quickly placed in liquid nitrogen and stored at −80 °C for biochemical and histological analyses.

### 4.2. Histological Analysis

Mouse tibialis anterior muscle was collected, and frozen sections were made according to the manufacturer’s instructions. Tissues were stained with hematoxylin and eosin (H&E), and paraffin sections were immunofluoresced with the CD31 antibody (Wuhan Servicebio Technology, Wuhan, China), then visualised using light microscopy.

### 4.3. Small RNA Sequencing

The extracted RNA underwent an initial pretreatment (Arraystar, Rockville, MD, USA) to eliminate modifications, such as 3′-carbamoyl, 2′,3′-cyclic phosphate, 5′-OH, m1A, and m3C. Subsequently, cDNA was synthesised and amplified using reverse transcription (RT) and amplification primers. The resulting cDNA fragments, ranging in size from 135 to 160 bp, corresponding to small RNA sizes of 15–40 nt, were purified using polyacrylamide gel electrophoresis (PAGE). The quality and quantity of the completed libraries were assessed using the 2100 Bioanalyzer (Agilent, Santa Clara, CA, USA). High-throughput sequencing was performed on the NextSeq 500 platform (Illumina, San Diego, CA, USA). The sequencing data were deposited in the Genome Sequence Archive (GSA) database with the identification number CRA007436.

### 4.4. Cell Culture and Treatment

The C166 vascular endothelial cells and 293T cells of mice were purchased from NICR (Beijing, China) and cultured in the DMEM high-sugar medium (meilunbio, Dalian, China), containing 10% of foetal bovine serum (FBS; NEWZERUM, Auckland, New Zealand), at 37 °C and 5% CO_2,_ in a humidified environment. During cell transfection, the Lipo3000 (Invitrogen, Carlsbad, CA, USA) transfection reagent was separately dissolved in an appropriate serum-free culture medium (Opti-MEM, Gibco, Scotland, UK) along with the small RNA at room temperature for 15 min. Subsequently, the two solutions were mixed and incubated at room temperature for another 15 min. The incubated mixture was then added dropwise onto the cell culture dish, ensuring even coverage of the entire cell layer. The specific sequence information is as follows: NC (5′ to 3′, UUGUACUACACAAAAGUACUG), tRF^GlnCTG^ mimics (5′ to 3′, UUGGUGUCAGGCUAGUUUU), si-NC (5′ to 3′, Sense: UUCUCCGAACGUGUCACGUTT, Antisense: ACGUGACACGUUCGGAGAATT), and si-Antxr1 (5′ to 3′, Sense: GGACAACUUUAAUGAAACUTT, Antisense: AGUUUCAUUAAAGUUGUCCTT).

### 4.5. Cell-Proliferation Assay

Following the cell transfection in a 96-well for 24 h, 10 μL of CCK-8 reagent (Yeasen, Shanghai, China) was added into each well and incubated for 1.5 h in a 37 °C incubator. Finally, the Varioskan LUX (Thermo Scientific, Singapore) was used to measure the absorbance at 450 nm. Cell proliferation was detected using a Cell-Light EdU Apollo567 In Vitro Kit (RiboBio, Guangzhou, China), following the manufacturer’s protocol. The cells were incubated with 50 mM of EDU solution at 37 °C for 2 h, fixed with 4% paraformaldehyde, and then stained with the Apollo Dye reaction and Hoechst stain. Finally, the cells were observed and imaged using a fluorescence microscope, and the fluorescence results were analysed using ImageJ 1.8 (USA). The flow cytometry experiment was performed as follows: the digested cells were fixed with 75% of ethanol at −20 °C for 18 h, washed and added to the fluorescent dye (BD Cycletest™ Plus DNA Reagent Kit, San Jose, CA, USA), and then assayed using a flow cytometer (BD FACSVerse^TM^, Piscataway, NJ, USA), and the measurements were analysed using Modfit 3.1.

### 4.6. RNA Extraction and Quantitative Real-Time PCR

The total RNA was purified from the cell and tissue samples using Trizol (Sigma, St. Louis, MO, USA) according to the manufacturer’s instructions, followed by the cDNA synthesis using a reverse transcriptase kit (Takara, Kyoto, Japan). The cDNA was measured using quantitative real-time PCR using the TaKaRa TB Green^®^ Fast qPCR Mix (Takara, Kyoto, Japan). The U6, GAPDH, and β-actin housekeeping genes were used to normalise the gene expression levels of target genes.

### 4.7. Western Blot Analysis

Western blot was used to detect protein expression. The cells were grown in six-well plates after 24 h of drug treatment; a cell lysate was added; the protein concentration was measured using the BCA method; a protein-loading buffer was added; this was boiled at 100 °C for 10 min, and was then followed by the SDS-PAGE electrophoresis, membrane transfer, and closure; the primary antibody CD31 (1:1000; Wuhan Servicebio Technology; P16284) and the Actin (1:2000; Wuhan Servicebio Technology; P62736) were incubated overnight at 4 °C, then eluted; the secondary antibody (1:3000; Wuhan Servicebio Technology; GB23204) was incubated at room temperature for l h, eluted again, and developed.

### 4.8. Wound-Healing Assay

The C166 cells were inoculated in six-well plates. Three separate wounds were scraped using a 200 μL pipette tip and the cells were rinsed with a culture medium. Photographs of the wounds were taken at 0 h and 24 h in the same positions under the microscope. The cell migration capacity was quantified and analysed using the Image Pro Plus software 7.0 (IPP, Media Cybernetics, Rockville, MD, USA).

### 4.9. Tube-Formation Assay

The Ceturegel^®^ Matrix High Concentration (Yeasen, Shanghai, China) was thawed, spread evenly onto 96-well plates, and incubated at 37 °C for 30 min; then, 3000–5000 cells were suspended in 50 µL of serum-free medium and spread on a matrix gel. The extent of test tube formation was assessed by quantifying the number of branch points in the same field of view using ImageJ after 4–6 h of seeding.

### 4.10. Dual-Luciferase Reporter Assay

The RNAhybrid database was used to identify the tRF^GlnCTG^’s binding sites to Antxr1. The Antxr1 wild-type (Antxr1-WT) and mutant (Antxr1-MUT) luciferase reporter plasmids were designed and synthesised by Biotech Bioengineering (Shanghai, China). The plasmid vector used was psiCHECK-2. The luciferase reporter vector and tRF^GlnCTG^ mimics or tRF^GlnCTG^ negative control (NC) were transfected into the 293T cells using Lipo3000. Twenty-four hours after transfection, the relative luciferase activity was measured using a dual-luciferase reporter gene assay kit (Promega, Madison, WI, USA).

### 4.11. Data Analysis

All cellular experimental results were repeated at least three times and the data were processed using GraphPad Prism 8.0 and expressed as mean ± standard deviation (SD). The data were analysed using the one-way analysis of variance (ANOVA), and then the results were analysed for significance using Student’s *t*-test: *p* < 0.05 was considered statistically significant.

## Data Availability

The corresponding author can be contacted if necessary.
